# Mutations at the Serine Hydroxymethyltransferase Impact Its Interaction with a Soluble NSF Attachment Protein and a Pathogenesis-Related Protein in Soybean

**DOI:** 10.3390/vaccines8030349

**Published:** 2020-06-30

**Authors:** Naoufal Lakhssassi, Sarbottam Piya, Dounya Knizia, Abdelhalim El Baze, Mallory A. Cullen, Jonas Meksem, Aicha Lakhssassi, Tarek Hewezi, Khalid Meksem

**Affiliations:** 1 Department of Plant, Soil and Agricultural Systems, Southern Illinois University, Carbondale, IL 62901, USA; naoufal.lakhssassi@gmail.com (N.L.); dounya.knizia@siu.edu (D.K.); abdelhalim.elbaze@siu.edu (A.E.B.); mallory.cullen@siu.edu (M.A.C.); 2 Department of Plant Sciences, University of Tennessee, Knoxville, TN 37996, USA; spiya@utk.edu (S.P.); thewezi@utk.edu (T.H.); 3 Trinity College of Arts and Sciences, Duke University, Durham, NC 27708, USA; jonas.meksem@duke.edu; 4 Faculty of Sciences and Technologies, University of Lorraine, 54000 Nancy, France; aicha.lakhssassi@gmail.com

**Keywords:** α-SNAP, SHMT, PR08-Bet VI, Peking, PI88788, SCN resistance, EMS mutagenesis, site directed mutagenesis, alanine scanning, mutational analysis, protein-protein interaction

## Abstract

Resistance to soybean cyst nematodes (SCN) in “Peking-type” resistance is bigenic, requiring *Rhg4-a* and *rhg1-a*. *Rhg4-a* encodes a serine hydroxymethyltransferase (GmSHMT08) and *rhg1-a* encodes a soluble NSF attachment protein (GmSNAP18). Recently, it has been shown that a pathogenesis-related protein, GmPR08-Bet VI, potentiates the interaction between GmSHMT08 and GmSNAP18. Mutational analysis using spontaneously occurring and ethyl methanesulfonate (EMS)-induced mutations was carried out to increase our knowledge of the interacting GmSHMT08/GmSNAP18/GmPR08-Bet VI multi-protein complex. Mutations affecting the GmSHMT08 protein structure (dimerization and tetramerization) and interaction sites with GmSNAP18 and GmPR08-Bet VI proteins were found to impact the multi-protein complex. Interestingly, mutations affecting the PLP/THF substrate binding and catalysis did not affect the multi-protein complex, although they resulted in increased susceptibility to SCN. Most importantly, GmSHMT08 and GmSNAP18 from PI88788 were shown to interact within the cell, being potentiated in the presence of GmPR08-Bet VI. In addition, we have shown the presence of incompatibility between the GmSNAP18 (*rhg1-b*) of PI88788 and GmSHMT08 (*Rhg4-a*) from Peking. Components of the reactive oxygen species (ROS) pathway were shown to be induced in the SCN incompatible reaction and were mapped to QTLs for resistance to SCN using different mapping populations.

## 1. Introduction

Soybean (Glycine max (L) Merr.), a valuable source of protein, nutritional oil, and biodiesel, is one of the most important crops worldwide [1,2,3]. However, soybean production is limited by soybean cyst nematodes (SCN), a microscopic roundworm that feeds on the roots of soybeans, causing over USD 1.2 billion in yield loss annually in the U.S. alone [4]. Planting of resistant cultivars is the main strategy to control this pathogen [5]. Increases in the virulence of SCN populations on most known resistant sources urges the need for understanding the SCN resistance pathway. Peking and PI88788 are considered the two major types of SCN resistance in soybean accessions [5]. Soybean cv. ‘Forrest’ is a Peking-type SCN-resistant line that requires both *rhg1-a* (GmSNAP18) [6,7] and *Rhg4-a* (GmSHMT08) [8,9,10] as the major resistant genes, as opposed to PI88788-type resistance that utilizes the rhg1-b allele of GmSNAP18, a wound-inducible domain protein (WI12), and an amino acid transporter [11,12,13]. EMS mutagenesis in Forrest provided important evidence for the identification and discovery of the major gene at the *Rhg4-a* locus (GmSHMT08) conferring resistance to SCN [8]. Copy number variation at the *rhg1* also plays a role in SCN resistance [11,14,15,16,17,18]. Recently, we demonstrated, through whole genome re-sequencing of 106 soybean lines, the impact of copy number variants at both the *rhg1* and *Rhg4* genes on broad-based resistance to SCN [18].

The serine hydroxymethyltransferase (SHMT) gene family is widely present in the plant and animal kingdoms. SHMT plays a role in one-carbon metabolism, methionine synthesis, and the maintenance of redox homeostasis during photorespiration [19,20,21]. Through a transaldimination reaction, the interconversion of serine/glycine and tetrahydrofolate (THF)/5,10-methyleneTHF is carried out by the SHMT enzyme [22]. In the glycine synthesis reaction, SHMT plays a major role by directing one-carbon units to the folate-mediated one-carbon metabolism that is required for methyl group biogenesis, nucleotide biosynthesis, and vitamin and amino acid metabolism [23]. In the serine synthesis reaction, SHMT is essential in the metabolic reactions of photorespiration, which is primordial for C3 plants. Through the glyoxylate cycle, SHMT plays a role in the maintenance of redox homeostasis, involving the gluthatione synthase and peroxidase genes. In plants, mutations at the mitochondrial AtSHMT1 cause a photorespiratory phenotype in Arabidopsis thaliana [24]. The mutation is due to a G→A transition at the 5′ splice site of the sixth intron of AtSHMT1, causing aberrant splicing and a premature translation termination [24]. In humans, mutations in the SHMT proteins were shown to be involved in cancers and cardiovascular diseases [25,26,27].

The soluble NSF attachment protein (SNAP) carries four tetratricopeptide repeat motifs, known as TPRs [6]. Proteins containing TPRs were proved essential determinants of signal transduction pathways responding to hormones such as ethylene, cytokinin, gibberellin, salicylate, and auxin [28,29], in addition to being involved in a plethora of cellular and molecular functions [30,31]. The molecular functions of TPR proteins include protein folding, transport, and transcriptional control [30,32,33]. TPR proteins are involved in several biological processes, such as cycle regulation, neurogenesis, mitochondrial, and peroxisomal protein transport [30]. TPR-containing proteins can be found in humans, yeast, bacteria, and plants. Mutations in TPR proteins produce several human diseases. In fact, mutations in the TPR containing protein aryl-hydrocarbon-interacting-protein-like 1 (AIPL1) results in Leber congenital amaurosis, one of the most severe inherited retinopathies [34]. Missense mutations in the TPR region of p67 phox, affecting TPR domain folding, have been implicated in chronic granulomatous disease [35]. Additionally, a TPR-Down (TPRD) protein was found to be involved in Down syndrome [36].

Recent experimental evidence pointed to a role of GmSHMT08 in DNA methylation [37,38]. In addition, a pathogenesis related protein, GmPR08-Bet VI, has been identified to physically interact with GmSHMT08 and plays a role in SCN resistance. Pathogenesis related proteins (PRs) are common in many viridiplantae and bind large hydrophobic compounds (i.e., lipids, hormones, and antibiotics). Some pathogenesis-related proteins are toxic to invading fungal pathogens. PRs are also involved in human diseases. It has been shown that the human glioma pathogenesis-related protein 1 (GLIPR1), a PR1 homologous gene, has tumor suppressor activities and is involved in the restoration of function in prostate cancer cells [39]. Similarly, loss of GLIPR1 function predisposed mice to tumorigenesis [40]. PRs can be secreted by the fusion of vesicles and SNAP proteins through translocation and docking at the plasma membrane, a process very well studied in animals and plants as well, involving the SNARE protein complex [41]. The SNARE complex involves several protein partners, including SNAP, PR, and other SNARE-related proteins and has been shown to contribute to gene for gene resistance against bacteria in *Nicotiana bentamiana* by secretion of PR1 in the extracellular space [42]. Overexpression of the pathogenesis-related protein AtPRP5 from *Arabidopsis thaliana* decreased SCN cyst number to less than 50% in transgenic soybean roots [43]. A PR10 protein was reported to play an important role in host defense against *Phytophthora sojae* infection [44].

In soybeans, a new crosstalk between the GmSHMT08 and GmSNAP18 proteins underlying SCN resistance in soybean was reported [29]. Both proteins interact at the molecular level, requiring the presence of another partner: the pathogenesis-related protein, GmPR08-Bet VI. Recent findings show that GmPR08-Bet VI transcripts were induced in response to SCN infections and its overexpression decreased the number of SCN cysts by nearly 65% in transgenic soybean roots [29]. In addition to the identified SCN resistant and SCN defense genes, the presence of a crosstalk has been suggested as involving the two phytohormones: salicylic acid and cytokinin [29]. The identification and discovery of the first step of the upstream SCN pathway involving the GmSHMT08/GmSNAP18/GmPR08-Bet VI multi-protein complex present an unprecedented plant resistance mechanism against a pathogen. In the current study, we reveal the impact of naturally occurring and induced mutations in GmSHMT08 and GmSNAP18 in the GmSHMT08/GmSNAP18/GmPR08-Bet VI multi-protein complex in Peking-type resistance. Unprecedently, this study investigates the presence of the GmSHMT08/GmSNAP18/GmPR08-Bet VI multi-protein complex in PI88788-type resistance. Taking into consideration the involvement of GmSHMT08, GmSNAP18, and GmPR08-Bet VI proteins in plants, animals, and human diseases including cancers, the findings revealed in this manuscript may have widespread implications within the field of biology and pharmacogenomics, paving the way for novel therapeutics.

## 2. Materials and Methods

### 2.1. Development of the EMS Mutagenesis Forrest Population

The wild type Forrest seeds, from Southern Illinois University Carbondale Agricultural Research Center, were mutagenized with 0.6% EMS as described by (Meksem et al., 2008). These seeds were planted to harvest M2 families, and advanced to the M3 generation at Southern Illinois University Carbondale as shown earlier [2].

### 2.2. Genotyping of ExF RIL Population

The ExF RIL population used in this study was developed at Southern Illinois University Carbondale [45]. The ExF genotyping was conducted as described by [6].

### 2.3. SCN-Infection Phenotyping

SCN screening was performed as described earlier [6,9].

### 2.4. Plasmid Construction for Y2H Analysis

The coding sequences of the *GmSNAP18* gene with only one (GmSNAP18^Δ73−184^), two (GmSNAP18^Δ109−184^), three (GmSNAP18^Δ152–184^), or four (full length) TPR domains were amplified from Forrest cDNA using forward and reverse primers containing *NdeI* and *SalI* restriction enzyme sites, respectively. The PCR product was digested and fused to the GAL4 DNA binding domain of the pGBKT7 bait vector (Clontech, Mountain View, CA 94043, USA). Similarly, the coding sequence of the *GmSHMT08* gene was PCR-amplified using forward and reverse primers containing *EcoRI* and *XhoI* restriction enzyme sites, respectively (Table S1). The PCR product was digested, purified and ligated to the GAL4 DNA activation domain of the pGADT7 prey vector (Clontech). All constructs were verified by sequencing.

### 2.5. Yeast Co-Transformation Assay

The pGADT7 prey construct containing the full length *GmSHMT08* coding sequence was transformed into *Saccharomyces cerevisiae* (yeast) strain AH109 together with various pGBKT7 bait plasmids. The co-transformed yeast cells containing bait and prey constructs were selected using SD/-Leu/-Trp medium. The interactions between GmSHMT08 and various GmSNAP18 deletions were identified by plating the co-transformed yeast cells onto the SD/-Leu-/Trp/-His and SD/-Leu/-Trp/-His/-Ade selective media. Serial dilutions of the co-transformed yeast cells were plated on the selective media to measure the strength of the interaction.

### 2.6. qRT-PCR Analysis

Soybean seedlings were grown in autoclaved sandy soil in a growth chamber for less than a week, and then infected with 2000 eggs from SCN HG-type 0 (race 3). Total RNA was isolated from the infected and non-infected root samples after three, five, and ten days following SCN infection as described previously [6]. The *GmSHMT08*, *GmSNAP18*, and *GmPR08-Bet VI* primers used for qRT-PCR have been described previously [29]. Experiments were repeated three times with similar results. Statistical analysis was performed using Student’s t-test for comparisons of means, using the JMP Pro V14 software (SAS Institute Inc., Cary, NC).

### 2.7. Protein Extraction and Co-Immunoprecipiation Analysis

Total proteins from Soybean Forrest-WT, Essex-WT, and the four recombinant inbred lines (RILs) were extracted in a lysis buffer containing 5 mM DTT, 1% (*v*/*v*) NP40, 1 mM sodium molybdate, 1 mM NaF, 1 mM PMSF, 1.5 mM Na_3_VO_4_, 100 mM NaCl, 2 mM EDTA, 50 mM Tris-HCl at pH 7.5, 10% (*v*/*v*) glycerol, and one tablet from the plant protease and phosphatase inhibitors at 1:100 mL (Thermo Scientific, Grand Island, NY 14072, USA). A Coomassie Bradford protein Assay Kit (Thermo Scientific) was used to quantify the protein concentration. For native gel analysis, DTT and SDS agents were removed. In planta co-IP analysis was performed as shown earlier [29].

### 2.8. BiFC Assay 

The coding sequence of Essex, Forrest, and PI88788 *GmSHMT08* wild type were cloned into *pSAT4-nEYFP-C1* as shown earlier [29]. The nine Gmshmt08 mutant alleles were cloned into *pSAT4-nEYFP-C1* to generate *nEYFP-GmSHMT08* mutant fusions (Additional Files 1–9). Likewise, *GmSNAP18* coding sequence from Essex, Forrest, and PI88788 were cloned into *pSAT4-cEYFP-C1-B*, as shown earlier [29]. The six *Gmsnap18* mutant alleles were cloned into *pSAT4-cEYFP-C1-B* to generate *cEYFP-GmSNAP18* mutant fusions (Additional Files 10–15). Various combinations of *cEYFP* and *nEYFP* fusions including controls were co-expressed in onion (*Allium cepa*) epidermal cells by particle bombardment as previously described [46] (Figure S2). In order to test the interactions among all genes, the *GmPR08-Bet VI* gene cloned into *pG2RNAi*, to generate *pG2RNAi2-GmPR08-Bet VI* fusions, was co-expressed along with *cEYFP* and *nEYFP* fusions in onion epidermal cells, as shown earlier [29]. Onion tissues co-transformed with cEYFP and nEYFP fusions were incubated in the dark at 25 °C, and after 16–36 h the tissues were examined for YFP activity. Fluorescent and bright field images were captured using the EVOS^®^ FL Auto Cell Imaging System (Life Technologies, Grand Island, NY 14072, USA).

### 2.9. Modeling of GmSNAP18, GmSNAP18, and GmPR08-Bet VI Proteins and Mutational Analysis

Homology modeling of putative GmSNAP18, GmSHMT08, and GmPR08-Bet VI protein structures was conducted using Deepview and Swiss-Model Workspace software as shown earlier [29]. Briefly, protein sequences from Forrest and available α-SNAP, SHMT, and PR crystal structures from *Rattus norvegicus* (PDB accession 3J96 chain G) [47], from *Homo sapiens* (1BJ4 chain A) [48], and from *A. thaliana* (2I9Y) were used as templates, respectively. Residues 6–284, 11-462, and 2–152 were modelled against their corresponding templates with a sequence identity of 39%, 60%, and 33% (according to the Protein Data Bank database). TPR domains, induced mutations, and haplotype mapping and visualizations were performed using the UCSF Chimera package [49]. To induce and map the corresponding naturally occurring and EMS-induced mutations and study their impact on the THF/PLP binding/catalysis, protein structure (dimerization and tetramerization), and the multi-protein complex interaction, the structural editing tool from the UCSF Chimera package was employed. Briefly, 5.0 Angstroms containing all atoms/bonds of any residue surrounding the mutated residue were selected first and shown in the model to study all possible residue interactions. Then, the rotamers tool that is incorporated within the Chimera package software was used to mutate the corresponding residues in order to study and predict their possible impact on protein activity and/or structure [50]. The rotamers tool allows amino acid sidechain rotamers to be viewed, evaluated, and incorporated into structures, where a given residue can be changed into different amino acids to predict the impact and effect of the mutations on the adjacent residues in a 5.0 Angstroms area surrounding the mutated residue. All three templates used met the minimum requirement of sequence homology (at least 30%) between the target and template [51].

### 2.10. Interaction Analysis of Homology Models

Interactions of the three-homology models including GmSNAP18, GmSNAP18, and GmPR08-Bet VI proteins were carried out as described earlier [29].

## 3. Results

### 3.1. GmSNAP18 and GmSHMT08 Interaction in Yeast

**Figure 1:**
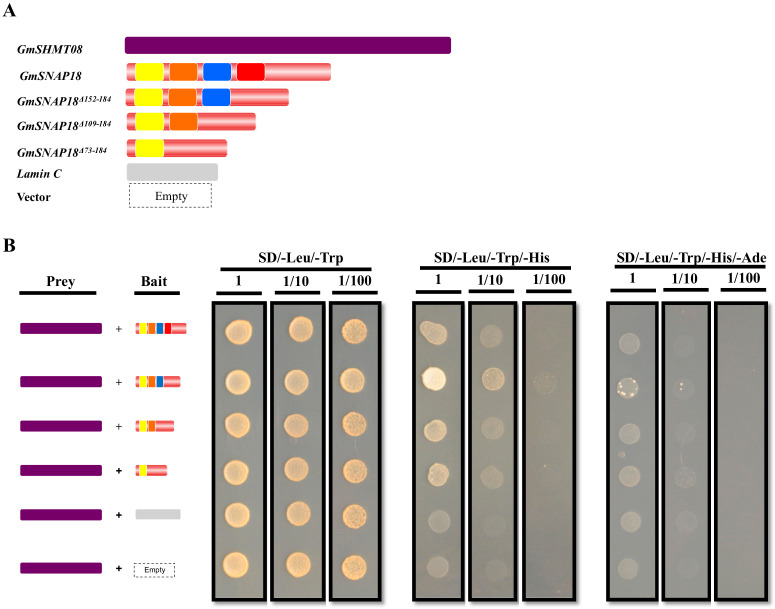
Impact of mutational analysis of the four TPR motifs GmSNAP18 interaction with GmSHMT08 in yeast. (**A**) Schematic structures of GmSHMT08 and GmSNAP18 constructs used in the yeast co-transformation assays. (**B**) Summary of yeast co-transformation assays. Yeast strain AH109 was co-transformed with *GmSHMT08* prey vector together with bait constructs containing one (*GmSNAP18^Δ73-184^*), two (*GmSNAP18^Δ109-184^*), three (*GmSNAP18^Δ152-184^*), or four (full length) TPR domains of GmSNAP18. Yeast cells containing bait and prey plasmids were selected by plating the cells on the SD/-Leu/-Trp medium. GmSNAP18/GmSHMT08 interaction were determined by differential growth on the selective SD/Leu/-Trp/-His and SD/-Leu/-Trp/-His/-Ade media. Empty bait plasmid and bait plasmid containing the human Lamin C gene were used as negative controls. Yellow, orange, blue, and red boxes represent TPR1, TPR2, TPR3, and TPR4 Tetratricopeptide repeat motifs at GmSNAP18 predicted protein, respectively. 1, 1/10, and 1/100 represent serial dilutions of the co-transformed yeast cells. The experiment was repeated three times and similar results were obtained.

GmSNAP18 contains four TPR motifs [6]. Proteins containing TPR domains facilitate specific interactions with partner proteins [30]. As shown earlier, protein homology modeling and docking algorithms have predicted the involvement of the TPR domain in the interaction with GmSHMT08 protein. In the current study, we first investigated the impact of TPR motif deletion on the interaction between the GmSHMT08 and GmSNAP18 proteins. Yeast co-transformation assays were used to examine the direct protein-protein interaction between the full coding sequence of GmSHMT08 and GmSNAP18 (used as a positive control), in addition to TPR deletions. In this assay, the coding sequences of *GmSNAP18* with only one (GmSNAP18^Δ73−184^), two (GmSNAP18^Δ109−184^), three (GmSNAP18^Δ152−184^) or four (full length) TPR motifs were amplified from Forrest cDNA and cloned into the bait vector as DNA-binding domain fusions (Figure 1A). Meanwhile, GmSHMT08 was cloned as a DNA-activation domain fusion in the prey vector. Yeast cells co-transformed with the *GmSHMT08* bait construct along with the prey *GmSNAP18* constructs were able to grow on the selective SD/-His/-Leu/-Trp medium (Figure 1B). Interestingly, while *GmSNAP18* constructs containing only one (GmSNAP18^Δ73−184^) or two (GmSNAP18^Δ109−184^) TPR motifs showed an interaction similar to that of the full-length gene, the *GmSNAP18* containing three (GmSNAP18^Δ152−184^) TPR motifs showed the strongest interaction (Figure 1B). No interaction was observed in yeast cells co-transformed with *GmSHMT08* and the empty bait vector or bait vector containing the human Lamin C gene (Figure 1B). When yeast co-transformed cells were selected on the stringent SD/-His/-Leu/-Trp/-Ade drop-out medium, only cells containing GmSHMT08 and GmSNAP18^Δ152−184^ showed weak interaction (Figure 1B). Together, these data strongly support the presence of a physical interaction between GmSHMT08 and GmSNAP18 that may require the presence of other partners. These results are coherent with recent findings showing the presence of a multi-protein complex within SCN infected root cells containing the GmSHMT08 and GmSNAP18, and requiring the newly identified partner GmPR08-Bet VI [29].

### 3.2. Resistant and Susceptible Alleles of GmSNAP18 and GmSHMT08 from Forrest and Essex can Physically Associate with Each Other

**Figure 2:**
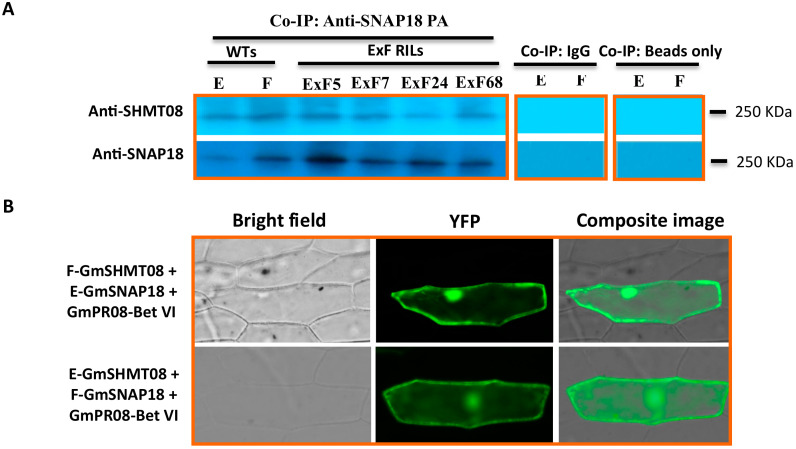
Interaction analyses of GmSNAP18 and GmSHMT08 proteins carrying resistant and susceptible allele combinations by Co-immunoprecipitation (Co-IP) and BiFC assay. (**A**) The total protein extracts of soybean Forrest, Essex, and four ExF RIL (Figure S1) roots were immunoprecipitated with Anti-GmSNAP18 PA. Blots from the eluted fraction were probed with both anti-SHMT08 and Anti-GmSNAP18. Upper bands on the panel (~ 250 KDa) correspond to the multi-protein complex including the tetrameric GmSHMT08 protein, lower bands on the panel (~ 250 KDa) correspond to the multi-protein complex including the GmSNAP18 protein. Native PAGE conditions (non denaturant) and western hybridization of the eluted fraction using both anti-GmSHMT08 and anti-GmSNAP18 antibodies showed the co-localization of the GmSHMT08 and GmSNAP18 binding. IgG and beads were used for Co-IP experiments as a negative control and technical control, respectively (**B**) BiFC analysis between GmSHMT08, GmSNAP18, and GmPR08-Bet VI proteins. The coding sequences of resistant Forrest (F) and susceptible Essex (E) alleles from *GmSNAP18* and *GmSHMT08* were cloned into *pSAT4-nEYFP-C1-B* and *pSAT4-cEYFP-C1* to generate nEYFP-SNAP18 and cEYFP-GmSHMT08 fusions, respectively. *GmPR08-Bet VI* was cloned into *pG2RNAi2*. Various combinations of cEYFP and nEYFP control fusions were co-expressed in onion epidermal cells by particle bombardment (Figure S2). Co-IP and BiFC assays indicated that resistant and susceptible alleles of GmSNAP18 and GmSHMT08 can associate each other.

We examined whether various haplotypes can impact the physical association between GmSHMT08 and GmSNAP18. To this end, co-immunoprecipitation analysis was conducted in six lines: the resistant Forrest-WT line and the susceptible Essex-WT line (both used as controls), in addition to the four ExF RILs carrying various combinations of the resistant and susceptible haplotypes (*GmSNAP18^+^/GmSHMT08^+^*, *GmSNAP18^−^/GmSHMT08^−^*, *GmSNAP18^+^/GmSHMT08^−^*, and *GmSNAP18^−^/GmSHMT08^+^*) under SCN infection (Figure S1). Proteins from the total soybean root extract were incubated in the presence of the immobilized anti-GmSNAP18 antibody. Under native PAGE conditions, western hybridization of the eluted fraction using anti-GmSHMT08 antibodies showed the presence of GmSHMT08 binding in Essex, Forrest, and the four ExF RILs analyzed (Figure 2A). These data support the idea that both GmSNAP18 and GmSHMT08 are components of the same protein complex in vivo.

Moreover, the presence of the GmSNAP18 protein in the same complex was confirmed using anti-GmSNAP18 antibodies in Essex, Forrest, and all of the RILs analyzed. These data show that both resistant and susceptible alleles of GmSNAP18 and GmSHMT08 can physically associate with each other. Interestingly, the obtained co-immunoprecipitation results are coherent with BiFC analysis, showing that susceptible E-GmSNAP18 interacts with resistant F-GmSHMT08 and vice-versa (F-GmSNAP18 interacts with E-GmSHMT08) (Figure 2B).

Most importantly, blotting tests with both anti-GmSHMT08 and anti-GmSNAP18 antibodies showed the presence of a protein complex of the same size corresponding to ~250 KDa (Native PAGE) (Figure 2A). These data suggest that in addition to a tetrameric GmSHMT08 protein (~200KDa) and GmSNAP18 molecule (~32 KDa), the other ~18 KDa may correspond to other protein interacting partner(s) that may be present in the same complex.

Notably, the calculated molecular mass of the immunoprecipitated pathogenesis-related protein was 17.76 KDa (theoretical PI of 5.96), which is the approximate size (~18 KDa) of the expected polypeptide partner that was suggested to be part of the GmSHMT08 and GmSNPA18 interacting protein complex (Figure 2A). These data support the previous results obtained from mass spectrometry and BiFC analysis showing the presence of the GmSNAP18/GmSHMT08/GmPR08-Bet VI multi-protein complex [29].

### 3.3. Expression and Interaction Analysis of GmSHMT08, GmSNAP18, and GmPR08-Bet VI in PI88788-Type Resistance

**Figure 3:**
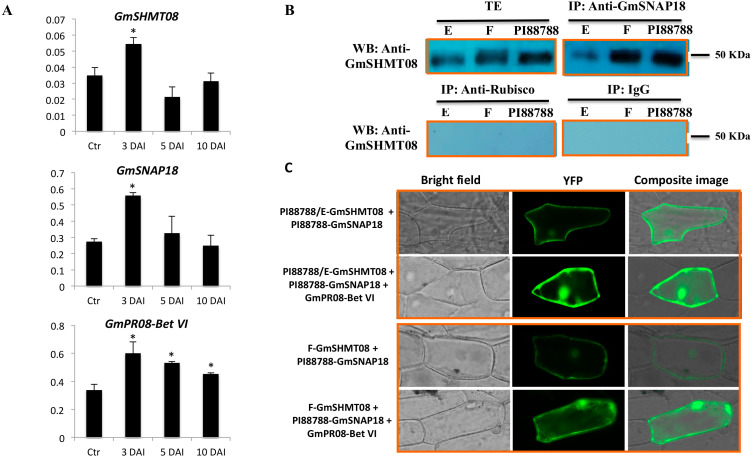
Expression and interaction analyses of GmSNAP18 and GmSHMT08 proteins carrying PI88788 alleles by Co-immunoprecipitation (Co-IP) and BiFC assay. (**A**) qRT-PCRanalysis of the GmSHMT08, GmSNAP18, and GmPR08-Bet VI genes in PI88788 from infected (3, 5, and 10 days) and non-infected (Ctr) root tissue with SCN HG-type 0. Expressions were normalized using Ubiquitin as reference. * Asterisks indicate significant differences between samples as determined by Student’s *t*-test (* p < 0.01). Error bars represent standard deviations. (**B**) The total protein extracts of soybean PI88788 roots were immunoprecipitated with Anti-GmSNAP18 PA. Forrest and Essex were used as positive control. Blots from the eluted fraction were probed with Anti-SHMT08. Western hybridization of the eluted fraction using both Anti-GmSHMT08 antibodies showed the presence of the GmSHMT08 and GmSNAP18 binding. IgG and Anti-Rubisco were used for Co-IP experiments as a negative control. (**C**) BiFC analysis between GmSHMT08, GmSNAP18, and/or GmPR08-Bet VI. The coding sequences of *GmSNAP18* and *GmSHMT08* were cloned into *pSAT4-nEYFP-C1-B* and *pSAT4-cEYFP-C1* to generate nEYFP-SNAP18 and cEYFP-GmSHMT08 fusions, respectively. *GmPR08-Bet VI* was cloned into pG2RNAi2. Various combinations of cEYFP and nEYFP control fusions were co-expressed in onion epidermal cells by particle bombardment (Figure S2). Co-IP and BiFC assays indicated that GmSNAP18 and GmSHMT08 alleles from PI88788 can associate each other.

To gain insight into the role of *GmSNAP18*, *GmSHMT08*, and *GmPR08-Bet VI* genes in soybean response to SCN infection, we analyzed their gene expression patterns in the roots of PI88788, in the absence and presence of SCN infection at 3, 5, and 10 days after infection (DAI). *GmSHMT08* and *GmSNAP18* transcripts were significantly induced in response to SCN infection at 3 DAI (early infection stage) (Figure 3A). Meanwhile, *GmPR08-Bet VI* transcripts showed a different expression pattern, being significantly induced at early, mid, and late SCN infection stages. The observed differences between resistance (*GmSNAP18* and *GmSHMT08*) and defense (*GmPR08-Bet VI*) genes may be related to the continuous role that *GmPR08-Bet VI* plays in reducing both exogenous and endogenous cytokinins at the feeding site, leading to cytokinin deficiency and therefore preventing syncytia expansion [29]. Regardless, all three genes respond to SCN infection in PI88788. Moreover, the obtained expression patterns are coherent with the copy number of *GmSNAP18*. In fact, Forrest, which carries three copies [18] of the *GmSNAP18*, was 3.23 times more abundant than in Essex (carrying 1 copy of the *GmSNAP18* [18]). On the other hand, PI88788, carrying nine copies [18] of the *GmSNAP18*, was 9.17 times more induced than Essex, at 3 days after SCN infection (Figure S3A). Clearly, the *GmSNAP18* copy number directly impacts its gene expression, playing a crucial role in plant resistance to SCN. 

It has been reported that the *GmSHMT08* in Peking-type resistance carries a resistant promoter (which is different from the susceptible promoter found in Essex and Williams 82), providing an additional layer of the SCN resistance mechanism [18]. As a result, *GmSHMT08* transcripts were 2.11 times more abundant in Forrest than in Essex at early SCN infection (Figure S3B). Similarly, PI88788 also carries the resistant *GmSHMT08* promoter [18], and its transcripts were 2.18 times more abundant when compared to Essex at the early SCN infection time point (Figure S3B). These data support the role of the resistant *GmSHMT08* promoter in inducing GmSHMT08 transcripts in response to SCN infections, which is consistent with the role of a tetrameric GmSHMT08 in resistance to SCN.

Interaction between the three proteins has been previously reported in Peking-type resistance; however, it is not clear if the GmSHMT08/GmSNAP18/GmPR08-Bet VI multi-protein complex is also present in PI88788. To test this hypothesis, two different approaches including co-immunoprecipitation analysis and BiFC assay were performed. Interestingly, co-immunoprecipiation analysis of the protein-eluted fraction from soybean root (SCN infected tissue) using anti-GmSNAP18 antibodies and blotted with anti-GmSHMT08 antibodies showed the presence of binding at 50 KDa (SDS PAGE), which corresponds to the GmSHMT08 homomers. Western blotting showed lower binding intensity in the SCN-susceptible Essex line when compared to the resistant PI88788 and Forrest lines. Notably, the presence of gene dosage effect is in agreement with the gene expression analysis and copy number shown above.

Additionally, BiFC assays supported the co-immunoprecipitation results and demonstrated that both GmSHMT08 and GmSNAP18 proteins associate with each other in PI88788, showing a stronger interaction in the presence of the GmPR08-Bet VI protein, similar to that of Peking-type resistance (Figure 3B). PI88788 carries the susceptible *GmSHMT08* allele, which is similar to Essex, but different from the resistant *GmSHMT08* allele in Forrest (Peking-type). When tested by BiFC, a weak signal was observed between the Forrest-GmSHMT08 and the PI88788-GmSNAP18. The presence of a weak interaction between two resistant alleles belonging to different types of resistance may be due to the presence of a haplotype compatibility and the presence of a different gene network. These results are supported by previous complementation analyses showing that the resistant *GmSNAP18^+^* Forrest haplotype was capable of restoring resistance to SCN in ExF-susceptible lines (carrying the susceptible *GmSNAP18^−^* Essex type and the resistant *GmSHMT08^+^* Forrest type), but not necessarily the resistant *GmSNAP18^+^* haplotype from PI88788 that was not capable of restoring resistance to SCN in the ExF susceptible lines [7]. Interestingly, this interaction was potentiated by the presence of GmPR08-Bet VI (Figure 3C). The presence of the multi-protein complex (GmSNAP18 at the *rhg1-b* locus, the GmSHMT08 at the *Rhg4-b* locus, and GmPR08-Bet VI) in PI88788 represents an unprecedented discovery, which may have widespread implications in breeding programs.

### 3.4. Mutational Analysis Supported the Predicted Interaction Model

Using both reverse and forward genetic approaches, we previously identified sixteen missense and two-nonsense EMS mutants at the GmSHMT08 protein [8,10]. SCN screening of all sixteen isolated mutants revealed a loss of resistance to SCN. Their female index increased significantly up to 93.4% (Figure S4). In the current study, we isolated a novel GmSHMT08_G357R_ carrying a missense mutation one residue away from the N358Y SNP found between Forrest and Essex, increasing the total of isolated Gmshmt08 EMS missense mutants to 17 (Figure S4). It has recently been reported that the Forrest-specific polymorphic substitution N358Y impacted the mobility of a loop near the entrance of the (6S)-tetrahydrofolate-binding site, severely reducing its affinity for folate and dramatically impairing enzyme activity in Forrest GmSHMT08 [52]. Interestingly, an EMS mutation at the Gly357 residue resulted in the highest increase in female index (FI > 113%) (Figure S4).

**Figure 4:**
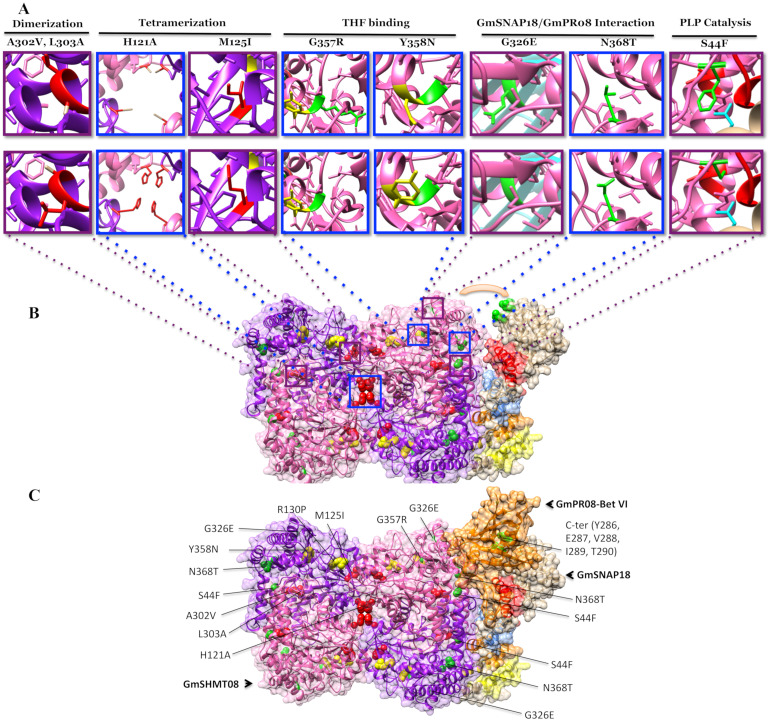
Mutational analysis supports the GmSNAP18/GmSHMT08/GmP08-Bet VI multi-protein complex predicted model. (**A**) The nine *Gmshmt08* mutant alleles used in the mutational analysis to study the predicted homology model (multi-protein). (down panel) represents the original residues in the Forrest WT, (up panel) represents the mutated residues (Induced and natural occurring mutations). (**B**) The predicted interaction between GmSHMT08 (left) and GmSNAP18 (Right). The surface in the middle (orange arc) correspond to the pocket where GmPR08-Bet VI protein was predicted to fit. Locations of the four TPR motifs (TPR1: Yellow, TPR2: Orange, TPR3: Blue, TPR4: Red) and polymorphisms (Green) at the GmSNAP18 are shown (Right). (**C**) The predicted interaction between GmSNAP18, GmSHMT08, and GmPR08-Bet VI protein complex. GmSHMT08 EMS induced mutations affecting Dimerization (red), Tetramerization (red), and Interaction (Green) with GmSNAP18 and GmPR08-Bet VI proteins are shown. The two polymorphisms R130P and Y358N between Essex and Forrest are shown in yellow. The GmSHMT08 EMS mutant M125I was identified earlier by TILLING (Liu et al., 2012); EMS mutants S44F, A302V, G326E, and N368T were identified by forward genetic (Kandoth et al., 2017). The EMS mutant G357R was identified by TILLING in this study. GmSHMT08^Δ+H121A^, GmSHMT08^ΔM125I^, GmSHMT08^Δ+L303A^, GmSNAP18^Δ+E208D^, GmSNAP18^Δ+Y286D^, GmSNAP18^Δ+E287D^, GmSNAP18^Δ+V288*^, and GmSNAP18^Δ+I289L^ mutations were produced by direct site mutagenesis in the current study and further tested their impact on the GmSHMT08/GmSNAP18/GmPR08 multi-protein complex complex by BiFC. The predicted interaction model was supported by BiFC analysis.

Out of the isolated 17 missense mutants, 5, 2, 2, 1, 4, and 1 *Gmshmt08* mutants were predicted to impact the THF binding, PLP binding, PLP catalysis, THF catalysis, GmSHMT08 dimerization, and GmSHMT08 tetramerization, respectively (Figure 4 and Figure S4). The remaining two *Gmshmt08* mutations were not located at the dimerization, tetramerization, or at the PLP/THF catalysis or binding sites. In fact, these two *Gmshmt08_G326E_* and *Gmshmt08_N368T_* mutations were mapped near to the interaction sites of GmSNAP18 and GmPR08-Bet VI. The newly identified *Gmshmt08_G357R_* mutant was mapped on the surface very close to the Y358N polymorphism.

**Figure 5:**
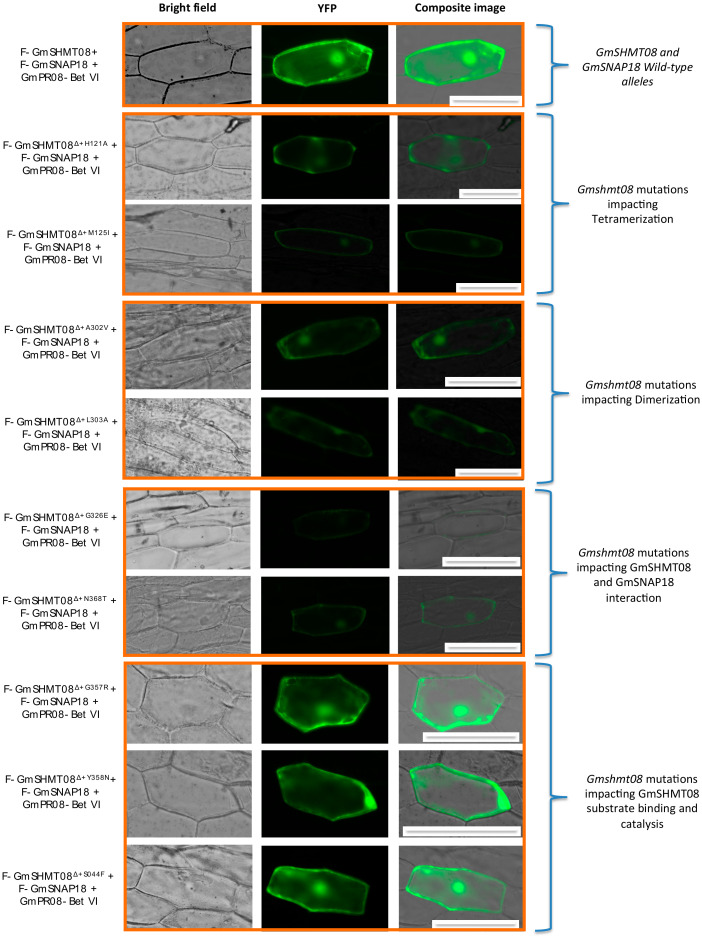
BiFC analysis between GmSNAP18, GmPR08, and the nine GmSHMT08 mutated alleles. The coding sequence of the 9 *GmSHMT08* mutant alleles were cloned into *pSAT4-nEYFP-C1* to generate *nEYFP-GmSHMT08* mutant fusions. Likewise, *GmSNAP18* from the Forrest WT and *GmPR08-Bet VI* were cloned into *pSAT4-cEYFP-C1-B* and *pG2RNAi2* to generate *cEYFP-GmSNAP18* and *pG2RNAi2-GmPR08-Bet VI* fusions. Various combinations of cEYFP and nEYFP control fusions were co-expressed in onion epidermal cells by particle bombardment (Figure S2). Bar = 200 µM.

In order to study the effect of the mutations at key residues of GmSHMT08, BiFC assays were carried out. Nine *Gmshmt08* mutants (*Gmshmt08^Δ+S44F^*, *Gmshmt08^Δ+H121A^*, *Gmshmt08^Δ+M125I^*, *Gmshmt08^Δ+A302V^*, *Gmshmt08^Δ+L303A^*, *Gmshmt08^Δ+G326E^*, *Gmshmt08^Δ+G357R^*, *Gmshmt08^Δ+Y358N^*, and *Gmshmt08^Δ+N368T^*) carrying mutations in residues located at the interaction site between all subunits constituting the GmSNAP18/GmSHMT08/GmPR08-Bet VI multi-protein complex, in addition to mutations affecting PLP/THF binding/catalysis, dimerization and tetramerization of the GmSHMT08 subunits, were included in the BiFC assay. All these mutations resulted in increased female indices, and consequently in a susceptible reaction toward the soybean cyst nematode (Figure S4). Interestingly, BiFC analysis shows that tested mutations affecting GmSHMT08 dimerization (*Gmshmt08^Δ+A302V^* and *Gmshmt08^Δ+L303A^*), tetramerization (Gm*shmt*08^Δ+H121A^ and Gm*shmt*08^Δ+M125I^), and the interaction with GmSNAP18 and GmPR08-Bet VI proteins (Gm*shmt*08^Δ+G326E^, and Gm*shmt*08^Δ+N368T^) negatively impacted the interaction of the GmSNAP18/GmSHMT08/GmPR08-Bet VI multi-protein complex. Interestingly, *Gmshmt08* mutations affecting the THF substrate binding (Gm*shmt*08^Δ+G357R^ and Gm*shmt*08^Δ+Y358N^) and PLP catalysis (Gm*shmt*08^Δ+S44F^) did not necessarily affect the GmSNAP18/GmSHMT08/GmPR08-Bet VI multi-protein complex, although they resulted in an increase in the SCN female index (Figure 5).

**Figure 6:**
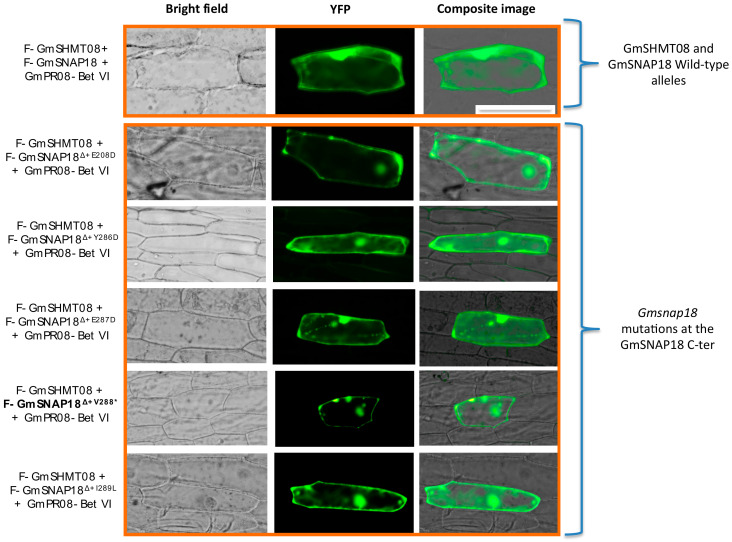
BiFC analysis between GmSHMT08, GmPR08, and the six GmSNAP18 mutant alleles. The coding sequence of the six *GmSNAP18* mutant alleles were cloned into *pSAT4-nEYFP-C1-B* to generate nEYFP-SNAP18 fusions. Likewise, *GmSHMT08* and *GmPR08-Bet VI* from the Forrest-WT were cloned into *pSAT4-cEYFP-C1* and *pG2RNAi2* to generate cEYFP-GmSHMT08 and cEYFP-GmPR08-Bet VI fusions. Various combinations of cEYFP and nEYFP control fusions were co-expressed in onion epidermal cells by particle bombardment (Figure S2). Bar = 200 µM.

Additionally, we tested, using BiFC, the five *Gmsnap18* mutations (*Gmsnap18^Δ+E208D^*, *Gmsnap18^Δ+Y286D^*, *Gmsnap18^Δ+E287D^*, Gmsnap18^Δ+V288*^, and *Gmsnap18^Δ+I289L^*) corresponding to the five naturally occurring mutations between the Essex and Forrest cultivars that are present at the C-terminal (Supplemental Figure S4B). The presence of these naturally occurring mutations did not impact the GmSNAP18/GmSHMT08/GmPR08-Bet VI multi-protein complex interactions (Figure 6).

### 3.5. Genes Encoding Key Components of ROS Signaling Pathway were Induced Under SCN Infection

**Figure 7:**
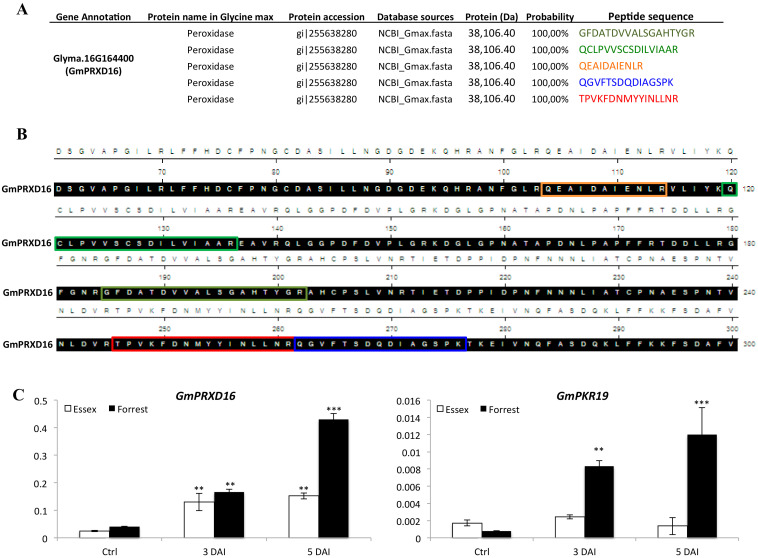
LC-MS protein identification of the eluted fraction obtained by immunoprecipitation using immobilized anti-SHMT08 antibodies and expression analysis. (**A**) Fragmented peptides identified by LC-MS in SCN infected root samples from Forrest. (**B**) Alignment of the GmPRXD16 protein sequence showing the five identified fragmented peptides by LC-MS. (**C**) Expression analysis of component of the ROS signaling pathway reveals that both genes are co-regulated in root cells undergoing nematode infection. Transcripts of genes encoding key components of the ROS signaling pathway including the peroxidase *GmPRXD16* and the protein kinase *GmPKR19* genes were induced and more abundant under SCN infection in the resistant line Forrest than in the susceptible line Essex. Asterisks indicate significant differences between the tested lines as determined by ANOVA (*** *p* < 0.001, ** *p* < 0.01, * *p* < 0.05).

Analysis of the five fragmented peptides obtained from the LC-MS analysis (Figure 7A) identified the presence of a peroxidase on Chromosome 16 (Glyma.16G164400), named GmPRXD16 (Figure 7B). In order to reveal the possible link of the H_2_O_2_ pathway in response to SCN infection, we analyzed the expression of the *GmPRXD16* gene in two lines: the susceptible line Essex and the resistant line Forrest, in the absence and presence of SCN infection at 2 and 5 days. The analysis showed that *GmPRXD16* transcripts were highly induced in the incompatible reaction (Figure 7C). Additionally, we tested the expression of the protein kinase *GmPKR19* gene, as it has been shown that protein kinases increase with reactive oxygen species (ROS) [53]. *GmPKR19* transcripts were significantly induced in the incompatible reaction at 3 and 5 days after SCN infection (Figure 7C).

## 4. Discussion

### 4.1. The Presence of a Tetrameric GmSHMT08 Protein within the Multi-Protein Complex

Using immobilized anti-GmSNAP18, co-immunoprecipitation analysis of protein-eluted fraction demonstrated the presence of binding at ~250 kDa in native-gel conditions after blotting using both anti-GmSNAP18 and anti-GmSHMT08, suggesting that a tetrameric GmSHMT08 (~200 kDa) interacts with a molecule of GmSNAP18 (~32 kDa) and another molecule of GmPR08-Bet VI (~18 kDa). These data are coherent with previous studies reporting that SHMTs in eukaryotes are found as asymmetric tetramers (Appaji Rao et al., 2003; Lakhssassi et al., 2019; Patil et al., 2019). Therefore, the biochemical analysis performed in this study supported the BiFC analysis and the predicted homology model reported earlier [29]. According to Renwick et al. (2019), the human cytosolic serine hydroxymethyltransferase forms a very tight dimer that comes together to form a loose tetramer [48]. Based on that, another hypothesis could be the formation of a dimer of GmSHMT08 associated with two GmSNAP18 and two GmPR08-Bet VI molecules (or a hexamer). However, in such a case, the total molecular weight would be around ~200 kDa, which does not match with the obtained 250 kDa band after bloating with both anti-GmSHMT08 and anti-GmSNAP18 antibodies.

Furthermore, it has been shown that the ScSHMT His134 residue is conserved only between eukaryotic SHMTs and is involved in the interaction of the two SHMT dimers (Tetramerization). In fact, mutants of sheep cytosolic Scshmt_His134_ have been found as dimers, suggesting that this area is involved in the tetramerization of SHMT. Any disruption of this area may lead to loss of tetramerization (Jagath et al., 1997a). Therefore, our data support the idea that, at least in soybean, the cytosolic GmSHMT08 is present as a tetramer, as has been suggested earlier [9,10,29,52]. Moreover, mutations affecting the GmSHMT08 subunits, including dimerization and tetramerization residues, negatively impacted the GmSNAP18/GmSHMT08/GmPR08-Bet VI multi-protein complex interaction, suggesting that the presence of the GmSHMT08 tetramer is essential for the multi-protein complex.

### 4.2. Mutational Analysis Reveals the Importance of the Gmshmt08 Tetrameric Structure in Maintaining the Multi-Protein Complex

Homology modeling and mutational analysis showed that the two L299F and A302V EMS mutations are localized in an α-helix involved in dimerization, and may affect the dimerization of two GmSHMT08 homomers to form a dimer. In *Escherichia coli*, a mostly nonpolar domain around residue eSHMT Leu276 (Forest Leu303) was involved in the dimerization of two eSHMT homomers [54]. The eshmt mutant L276A caused an alteration in the dimer-monomer equilibrium, resulting in mostly a monomeric eSHMT, while retaining the monomeric tertiary structure [54]. Our BiFC analysis demonstrated that GmSHMT08 mutations at the A302V and L303A residues negatively impacted the GmSHMT08/GmSNAP18/GmPR08-Bet VI multi-protein complex (Figure 4A). Thus, mutations in this α-helix may interrupt GmSHMT08 dimerization and therefore the presence of a tetramer impacting the formation of multi-protein complex.

Furthermore, the *Gmshmt08_M125I_* mutant is found near the conserved His121 (ScSHMT His134), which is conserved only between eukaryotic SHMTs and is involved in the interaction of the two dimers (Tetramerization). The SHMT enzyme exists in homotetrameric or homodimeric form, the latter being the minimum formation necessary for its catalytic function. Mutants of sheep cytosolic (ScSHMT) His134 have been found as dimers, suggesting that this area is involved in the tetramerization of SHMT. Any disruption of this area may lead to loss of tetramerization [55]. In the current study, mutations in both H121A and M125I residues at the GmSHMT08 negatively impacted the GmSHMT08/GmSNAP18/GmPR08-Bet VI multi-protein complex interaction, reinforcing the hypothesis that a GmSHMT08 tetramer is essential for the multi-protein complex (Figure 4A). The presence of this structure may explain the reported gene dosage effect, high copy number, and the induced expression of the *GmSHMT08* in resistant lines (carrying the resistant *GmSHMT08* promoter) [18,29]. The presence of a tetrameric structure of the GmSHMT08 protein is also consistent with soybean lines containing high copy numbers (up to 4) of the *GmSHMT08*, resulting in broad based resistance to SCN [18].

### 4.3. Induced and Natural Gmshmt08 Mutations at the PLP Catalysis and THF Substrate Binding Result in SCN Susceptibility but not Necessary Impacting the Multi-Protein Complex Interactions

The E61K EMS mutation is predicted to impact cofactor binding at the GmSHMT08 catalytic site (Figure 4A). It has been shown that Glu74 in sheep liver cytosolic SHMT (ScSHMT), corresponding to the *Gmshmt08_E61K_* mutant, catalyzes the cleavage of serine, thereby facilitating the L-Ser-geminal diamine to the external aldimine. A mutation at this site has been shown to affect the geometry of the active site and caused a 28-fold decrease in catalytic efficiency [56]. An *Scshmt_E74K_* mutant was proven to lose activity due to an inability to undergo a conformational change after binding to L-serine [57]. Our *Gmshmt08_E61K_* is likely to have the same conformational deficiency. These results are coherent with the in vitro kinetic studies of the *Gmshmt08* mutated alleles E61K and G71D when compared to the Forrest GmSHMT08 allele (Liu et al., 2012; Kandoth et al., 2017). In fact, enzymatic properties of the GmSHMT08 alleles carrying the E61K and G71D mutations resulted in proteins that are enzymatically inactive, as they were unable to support the growth of the bacteria, while the GmSHMT08 allele from the wild type Forrest supported growth of the mutant bacteria (Liu et al., 2012; Kandoth et al., 2017).

Additionally, E61K is found two residues away from the required Tyr59, which establishes a cation –π interaction with Arg250 and acts as an acid-base catalyst and hydrogen exchanger in the transaldimination process. Disruption of this site has been shown to profoundly alter substrate binding and catalytic activity in E. coli [58]. The Gmshmt08 mutant R257Q is located on the same β-strand of the essential Arg250 at the dimerization site, which also holds an arm protruding out of GmSHMT08 (Figure 4A).

The Forrest polymorphism R130P and *Gmshmt08_G132D_* mutant are both found near the site of two essential and conserved histidine residues: His134 and His137 (corresponding to His147 and His150 in scSHMT). The ScSHMT His147 has been shown to be required for the cofactor binding of PLP, and ScSHMT His150 is the base that abstracts the α-proton of the glycine external aldimine complex, which leads to a quinonoid intermediate [55,59]. The *E. coli* eSHMT Gly124 (Forrest Gly132) is involved in a stacking interaction between the PLP pyridine ring and eSHMT His126 (ScSHMT His147, GmSHMT His134) [58]. The presence of this new haplotype may be the reason why *Gmshmt08_G132D_* lost its catalytic activity, and could explain why the Forrest haplotype R130P is less catalytically active than the Essex Pro130 haplotype [8,52]. Considering proline has a conformational rigidity due to its direct incorporation of the α-carbon into its side chain, this may cause drastic conformational changes, interfering with this catalysis (Figure 4). Because the *Gmshmt08_G132D_* mutant was located between residues involved in THF binding (Leu129 and Gly133) and next to an important catalytic residue (His134), the G132D mutation is predicted to impact the THF binding site or catalysis.

It has been suggested that polymorphism residues that reside near to the ligand-binding sites may impair a key regulatory property of the GmSHMT08 enzyme [8]. In fact, the two Forrest polymorphic substitutions (P130R and N358Y) impact the mobility of a loop near the entrance of the THF-binding site at the GmSHMT08 protein, resulting in reduced affinity for folate substrate, subsequently impairing the enzymatic activity of GmSHMT08 [52]. The isolated novel Gmshmt08 mutant from the EMS mutagenized Forrest soybean population G357R (FI = 113%) is located one residue away from the Forrest polymorphic substitution N358Y, and, therefore, is predicted to impact the THF site’s binding to folate. Another mutation, S44F, which resulted in a loss of SCN resistance in Forrest, was mapped close to Tyr59 residues involved in PLP catalysis.

Unlike mutations affecting the dimerization and tetramerization of the GmSHMT08 protein, the increase in the SCN female index in the S44F, G357R, and Y358N *Gmshmt08* mutants is due to the loss of PLP catalysis and/or THF substrate binding [52]. These findings imply the role of both the GmSHMT08 structure and its enzymatic catalysis in SCN resistance. 

### 4.4. Mutations at GmSHMT08 Residues Mapped at the GmSNAP18/GmPR08-Bet VI Interacting Sites Negatively Impacted the Multi-Protein Complex 

Two *Gmshmt08* mutants, F1460 (G326E) and F1801 (N368T), were found to impact the interaction between GmSHMT08 and GmPR08-Bet VI (Figure 4). The induced missense mutation G326E changes the amino acid from a small non-polar R-group to the large negatively-charged glutamic acid, which could cause a steric hindrance at the interaction site, interrupting the α-helix that interacts with GmSNAP18/GmPR08-Bet V (Figure 4A,C). This may disrupt the interaction interface between the multi-protein complex. In the case of the N368T mutation, assuming that this mutation would not alter the tertiary structure of the expressed GmSHMT08 protein, the shift from a large asparagine to a smaller threonine may cause a steric effect on two α-helices at the interface with GmSNAP18/GmPR08-Bet V (Figure 4A,C). This may disrupt the interaction interface between the multi-protein complex. When tested by BiFC, both G326E and N368T mutations negatively impacted the interaction of the multi-protein complex, reinforcing the proposed interaction model.

### 4.5. GmSNAP18 C-Terminal Involvement in Driving the Multi-Protein Complex Toward SCN Infected Sites

The *GmSNAP18* gene in Forrest is present with three copies, and hence the probability of introducing EMS mutations in all three copies simultaneously is very slim. Therefore, we focused on naturally existing mutations between different GmSNAP18 haplotypes. Unlike mutations in the GmSHMT08 protein, mutations in the GmSNAP18 protein did not have a negative effect on the multi-protein complex interactions. Two main regions were found between SCN-susceptible Essex and SCN-resistant Forrest. The first region includes the Q208D haplotype (Figure 4B), whereas the second region involves the GmSNAP18 C-terminal (E285, Y286, E287, V288, and I289) (Figure 4B), that was suggested to alter the destination of a GmSNAP18-guided vesicle [7,60]. This was based on findings showing the involvement of the C-terminal region of SNAP proteins in determining its localization and function controlling vesicle trafficking and fusion [61]. The presence of the GmSNAP18 in the multi-protein complex may drive the complex to an altered destination, in light of the recent finding that rhg1 mediates SCN disease resistance through impairment of α-SNAP–NSF interaction and vesicular trafficking [12]. This is also coherent with in-situ and immunostaining analysis showing the hyper-accumulation of GmSNAP18 proteins at the plasma membrane of soybean root cells surrounding the nematode in SCN-resistant soybean lines [29,62]. The *GmSHMT08* promoter-GUS analysis in Forrest was also shown to be expressed in syncytial feeding cells at 3 DAI [8], which is coherent with the previous hypothesis involving GmSNAP18 in driving the multi-protein complex toward the SCN infection site.

### 4.6. GmSHMT08 as Mediator of Peking-Type SCN Resistance

**Figure 8:**
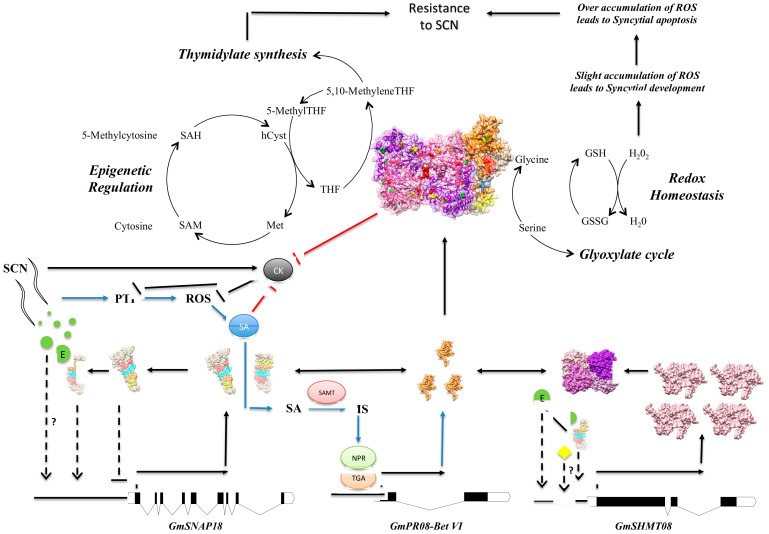
Deciphering SCN resistance mechanism in Peking-type. Cross-talk between SCN resistant genes, defense genes, and phytohormones is shown as described earlier (Lakhssassi et al., 2020). Dashed lines represent unknown possible intermediary steps. The possible binding of a nematode effector (E) may interrupt the autoregulation of GmSNAP18, causing an increase in the transcription of GmSNAP18, consequently causing an induction of GmSHMT08. Next, the multi-protein complex may modulate the activity of the GmSHMT08 in single carbon metabolism, methionine synthesis, and maintenance of redox homeostasis within the root cells. Induction of the apoptosis, necrosis, and degeneration observed in the cells surrounding the syncytia may occur. Metabolite abbreviations are as follows—(SAH) S-adenosylhomocysteine; (SAM) S-adenosylmethionine; (hCyst) homocysteine; (Met) methionine; (THF) tetrahydrofolate; (GSH) reduced glutathione; (GSSG) oxidized glutathione.

Taken together, the recent findings increase our understanding of the SCN resistance mechanism. First, recognition between nematode effectors (i.e., HgSLP-1) and resistant soybean lines is essential to trigger the incompatible interaction [29,63,64]. Binding of nematode effectors [65] may interrupt the negative autoregulation of GmSNAP18, increasing its transcription at the cellular level, which positively impacts the induction of GmSHMT08 transcripts. Increased salicylic acid induces salicylic acid defense genes, including *GmSAMT*, *GmNPR*, and *GmTGA* resulting in the induction of the *GmPR08-Bet VI* [29], favoring the multi-protein complex formation. Next, the presence of the GmSHMT08/GmSNAP18/GmPR08-Bet VI multi-protein complex may modulate the activity of the GmSHMT08 in the maintenance of redox homeostasis within the root cells, but may also affect the molecular trafficking of the GmPR08-Bet VI in the infected soybean roots increasing cytotoxicity in the cells surrounding the nematode to disrupt syncytium viability [29] (Figure 8). The presence of the GmSNAP18/GmSHMT08/GmPR08-Bet VI multi-protein complex within the cell dramatically intensified cell death and necrosis [29]. Since SHMT is involved in the simultaneous interconversion of serine/glycine and THF/5,10-methyleneTHF [66], we hypothesize that a potential modulation of its activity may cause a disruption of serine/glycine and/or THF/5,10-methyleneTHF interconversion.

On the serine/glycine side, the SHMT interconversion of serine/glycine may impact the maintenance of redox homeostasis that occurs via glutathione synthase and glutathione peroxidases. Another enzyme, glutathione S-transferases (GSTs), is known for its ability to catalyze the conjugation of the reduced form of glutathione (GSH) to xenobiotic substrates for detoxification [67,68,69]. The activity of GSTs is dependent upon GSH supply from the synthetic enzyme glutathione synthetase and the action of some transporters to remove GSH conjugates from the cell [70,71]. Glutathione peroxidases and glutathione transferase of τ-GST gene family transcriptions were significantly modulated in transcriptomic analysis of SCN infection (syncytia) among other ROS scavenging enzymes [72]. In fact, while the maintenance of ROS homeostasis at low levels is required for parasitic nematodes to cause pathogenic disease, disruption of this homeostasis (over accumulation of ROS) can lead to syncytial apoptosis (Figure 8) [73,74,75]. In the current study, two components of the ROS pathway were shown to be highly induced in the incompatible reaction (resistant lines) under SCN infection (Figure S5). Additionally, a peroxidase (*GmPRXD16*) and protein kinase receptor (*GmPKR19*) were mapped to QTLs for resistance to SCN [76,77] using different mapping populations (Figure S5). Similarly, genes belonging to the two glutathione transferases (*GmGST07* and *GmGST15*) and a glutathione peroxidase (*GmGPRXD08*) that were significantly modulated in transcriptomic analysis of SCN infection (syncytia) [72] were also mapped to QTLs for resistance to SCN [77,78,79]. In this context, overexpression of Arabidopsis peroxidase *AtPRX53* was found to influence plant susceptibility to the cyst *Heterodera schachtii* [80].

Moreover, the link between SHMTs, PRs, ROS and SA pathways has been established previously. In Arabidopsis, *Atshmt1-1* mutants showed a greater accumulation of H_2_O_2_, which is known to induce SA biosynthesis [81,82]. In soybeans, the induction of the SA pathway results in the induction of *GmPR08-Bet VI* following nematode infection [29]. The accumulation of H_2_O_2_ leading to SA induction and GmPR08-Bet VI accumulation in soybean-infected roots is believed to eventually result in cell death and necrosis, favoring syncytial apoptosis (Figure 8). 

On the THF side, 5,10-methyleneTHF and the nucleotide deoxyuridine monophosphate (dUMP) are both utilized by thymidylate synthetase to synthesize deoxythymidine monophosphate (dTMP). It has been demonstrated that *shmt* knockdown mutants induce apoptosis in lung cancer cells through the inhibition of thymidylate synthesis and consequent overabundance of uracil, therefore causing uracil misincorporation [83]. The 5,10-methyleneTHF is also found to be utilized by the S-adenosyl methionine (SAM) cycle in the synthesis of methionine. The SAM cycle is known to provide DNA methyltransferases with methyl groups from SAM [84]. Modulation of this cycle can result in differential activity of DNA methyltransferase, and may cause a shift in the epigenetic profile of the soybean roots during syncytial formation, as it has been shown that susceptible soybeans under SCN infection undergo a major change in their methylome [38,85]. Importantly, genome-wide analysis of DNA methylation patterns in two near-isogenic lines (NILs) differing at the GmSHMT08 locus pointed to a role of GmSHMT08 in methylome reprograming [37]. This mode of action reveals a new mechanism through which the induction of the reported apoptosis, necrosis, and degeneration observed in the cells surrounding the syncytia may occur [5,72,86].

Several molecular partners have been identified to interact with SNAP proteins in both animals and plants [12,87,88,89,90]. The discovery of a serine hydroxymethyltransferase and a pathogenesis-related protein as novel partners of the soluble NSF attachment protein in PI88788 and Peking-types of SCN resistance provide plants with a new response mechanism toward biotic stresses. Since the three proteins were reported to be involved in human diseases, this discovery may impact the fields of pharmacology, biomedicine, and other related disciplines.

## 5. Conclusions

The current study revealed the importance of a Tetrameric form of the GmSHMT08 Protein within the Multi-Protein complex needed for resistance to SCN. Unlike what was reported earlier in PI88788 about the requirement of only needing the *rhg1-b* for resistance to SCN, this study showed for the first time an interaction within the cell between *GmSNAP18* at the *rhg1-b* and GmSHMT08 at the *Rhg4-b* locus in the PI88788 type of resistance. Similarly to Peking-type resistance, the reported interaction between the *rhg1-b* and *Rhg4-b* in PI88788 was stronger in the presence of the GmPR08-Bet VI. Furthermore, BiFC analyses have confirmed the presence of haplotype compatibility between the *Rhg4-a* and *rhg1-a*, and between the *Rhg4-b* and the *rhg1-b,* reinforcing previous complementation analyses showing that resistant *GmSNAP18^+^* Forrest haplotype was capable of restoring resistance to SCN in ExF-susceptible lines (carrying the susceptible *GmSNAP18^−^* Essex type and the resistant *GmSHMT08^+^* Forrest type), but not the resistant *GmSNAP18^+^* haplotype from PI88788 that was not capable of restoring resistance to SCN in the ExF susceptible lines. The presence of the incompatibility between the GmSNAP18 (*rhg1-b*) of PI88788 and GmSHMT08 (*Rhg4-a*) from Peking may explain the difficulty of breeding for soybean lines that combine both the PI88788 and Peking type of SCN resistance. Most importantly, we have shown a clear presence of gene dosage effect impacted by the induction of the 3 genes (*GmSHMT08, GmSNAP18*, and *GmPR08-Bet VI*) in both Peking and PI88788 types of resistance, in addition to the presence of the resistant promoter at the *GmSHMT08*, and a high copy number of the *GmSNAP18*, positively impacting resistance to SCN in soybean cultivars. 

## Supplementary Materials

The following are available online at https://www.mdpi.com/2076-393X/8/3/349/s1, Figure S1: (A) Genotypes of the four ExF RIL populations used for the Co-IP analysis. (B) Female index of Essex, Forrest, and the four different ExF genotypes (*n* = 25), Figure S2: Negative controls of the BiFC analysis. Each of the cloned GmSNAP18 and GmSHMT08 in the pSAT4-nEYFP-C1 (E81) were tested in the presence of the *pSAT4-cEYFP-C1-B* (E82) and/or *pG2RNAi2* empty vectors used in the BiFC analysis in Figure 2, Figure 3, Figure 6 and Figure 7. Various combinations of cEYFP and nEYFP control fusions were co-expressed in onion epidermal cells by particle bombardment under the same conditions and experiments, Figure S3: qRT-PCR analysis of the *GmSHMT08*, *GmSNAP18*, and *GmPR08-Bet VI* genes in Essex, Forrest, and PI88788 from infected (3, 5, and 10 days) and non-infected (Ctr) root tissue with SCN HG-type 0. Expressions were normalized using Ubiquitin as reference. Asterisks indicate significant differences between samples as determined by Student’s t-test (** *p* < 0.01, * *p* < 0.05). Error bars represent Standard deviations, Figure S4. The isolated EMS missense *Gmshmt08* mutants. (A) GmSHMT08 gene model in Forrest wild type. (B) *GmSHMT08* polymorphisms between Forrest and Essex wild types, and the 17 missense *Gmshmt08* mutants identified by reverse ^a^ and forward ^b,c,d^ genetic approaches. PROVEAN predictions of the mutations identified were calculated using the PROVEAN software available at http://provean.jcvi.org. The primers used for TILLING and gDNA sequencing are indicated by arrows. FI: female index of soybean cyst nematode (SCN) after 30 days infection, lines with FI lower than 10% are considered to be resistant to SCN, *n* > 5. GmSHMT08 residues involved in THF and/or PLP cofactor binding and/or catalysis, dimerization/tetramerization of the GmSHMT08 protein, or affecting the multi-protein GmSHMT08/GmSNAP18/GmPR08-Bet VI complex subunits are shown. ^a^ Identified by TILLING (Liu et al., 2012). ^b^ Identified by forward genetics (Kandoth et al., 2017). ^c^ identified by forward genetics (Lakhssassi et al., 2019). ^d^ Identified by forward genetics in the current study, Figure S5. Physical positions corresponding to the peroxidase (*GmPRXD16*), the protein kinase receptor (*GmPKR19*), the two Glutathione transferases (*GmGST07* and *GmGST15*), and the Glutathione peroxidase (*GmGPRXD08*), and the identified SCN QTLs are shown; Glyma.07G140400 (Chr07: 16,641,129–16,643,278) and Glyma.07G139700 (Chr07: 16,580,992–16,582,517) were mapped within the QTL: SCN 3-3 (Gm07: 16,521,027 Mb-Gm07: 18,352,609 Mb) (Webb et al., 1995), Glyma08g05200 (Chr08: 3,685,159–3,688,493) was mapped 0.31 Mb away from the QTL: SCN 33-2 (Gm08: 3,993,698 Mb-Gm08: 8,223,512 Mb) (Guo et al., 2006), Glyma.15G251500 (Chr15: 47,176,586–47,178,221) was mapped within the QTL: SCN 25-1 (Gm15: 17,019,921 Mb–Gm15: 51,294,894 Mb) (Yue et al., 2001), Glyma.16G164400 (Chr16: 32,320,351–32,323,318) was mapped within the QTL: SCN 38-3 (Gm16: 30,404,629 Mb–Gm16: 33,818,897 Mb) (Chang et al., 2011), and Glyma.19G193100 (Chr19: 45,084,723–45,089,886) was mapped 0.02 Mb away from the QTL: SCN 29-7 (Gm19: 45,182,458 Mb–Gm19: 47,378,001 Mb) (Guo et al., 2006), Table S1: The primers used for genotyping, sequencing and subcloning, and yeast two-hybrid.
